# A systematic review of nature-based solutions for climate action in African cities

**DOI:** 10.1088/1748-9326/aeb0ba

**Published:** 2026-10-20

**Authors:** Nicola Frances Rule, Cameron T McLean, Andrew Emmanuel Okem, Promise M Nduku, Johannes Bhanye, Collins Matiza, Fred Sato, Chipo Perseverance Mungenge, Onyekachi Esther Ogunniyi, Ignacio Pita-Vaca, Cherié Janine Forbes, Petra Holden, Christopher H Trisos, Debra C Roberts, Nicholas P Simpson

**Affiliations:** 1 African Centre for Cities, University of Cape Town, Cape Town, South Africa; 2 African Climate and Development Initiative, University of Cape Town, Cape Town, South Africa; 3 Biodiversity Management Department, eThekwini Municipality, Durban, South Africa; 4 Deltares, Delft, The Netherlands; 5 School of Social Sciences, University of KwaZulu-Natal, Durban, South Africa; 6 African Synthesis Centre for Climate Change, Environment and Development (ASCEND), African Climate & Development Initiative, University of Cape Town, Cape Town, South Africa; 7 Climate Risk Lab, African Climate & Development Initiative, University of Cape Town, Cape Town, South Africa; 8 People in Nature & Climate Lab, African Climate & Development Initiative, University of Cape Town, Cape Town, South Africa; 9 South African National Biodiversity Institute, Kirstenbosch National Botanical Gardens, Newlands, Cape Town, South Africa; 10 School of Life Sciences, University of KwaZulu-Natal, Durban, South Africa; 11 Faculty of Geo-Information Science and Earth Observation, University of Twente, Enschede, The Netherlands; 12 School of Agriculture and Science, University of KwaZulu-Natal, Westville, South Africa

**Keywords:** ecosystem-based adaptation, climate change, urban governance, mitigation, informal settlements

## Abstract

Nature-based solutions (NbS), including ecosystem-based adaptation, are increasingly proposed for climate action in African cities, but the evidence base is fragmented and lacks systematic synthesis of what is being implemented, where, and how effectively. We analysed 112 studies retained from a systematic review, covering 134 urban areas across 42 countries and including 45 informal settlements. Five NbS typology classes are inductively derived including: Green spaces; Rivers, catchments and drainage systems; Coastal systems; Green buildings; and Urban agriculture and multi-use gardens. Urban green spaces dominate the evidence (49% of studies) and are the most multi-functional class. Urban agriculture and multi-use gardens are under-recognised in headline framings, named as primary in only 5% of studies but appearing in 34% of the wider intervention mix. Evidence is increasing but concentrates geographically on larger cities. Successful climate change adaptation and mitigation outcomes are reported by 72% and 27% of studies, respectively. Heat mitigation is the most frequently quantified adaptation outcome, with temperature reductions up to 12.2 °C reported in proximity to urban green and blue spaces across the continent. Qualitative evidence links urban NbS to effective coastal protection, flood risk reduction, and increased water security. Multi-stakeholder governance anchored by local actors, and meaningful community participation are consistent enablers of effective implementation. However, insufficient long-term funding and livelihood disruptions and displacement raise concerns about long-term impacts. Priority research gaps include longitudinal effectiveness monitoring, financing mechanisms for informal contexts, systematic assessment of mechanisms behind inequitable outcomes, and evidence from under-represented regions and smaller cities.

## 1. Introduction

Africa’s urban population is growing faster than in any other region [1]. The continent’s rapidly growing urban areas are highly susceptible to climate risk, where climate change amplifies pre-existing stresses related to poverty, informality, infrastructure deficits, and social and economic exclusion [2]. In addition, much of Africa’s population growth will continue to occur in informal settlements, where, despite often strong informal institutions and economies, risks to health, livelihoods and safety are compounded by ecosystem degradation, service delivery deficits, and concentrations of climate hazards like flooding [3–5]. Beyond concentrating risk, African cities also offer scalable opportunities for adaptation and mitigation [6]. Where governments fall short of universal service delivery, residents often cope through locally governed, hybrid systems that integrate conventional grey infrastructure with local and nature-based alternatives [7]. Some systems, such as grey-green drainage, offer opportunities for experimentation to manage climate uncertainty [8]. In contrast, some coping practices and systems to address centralised infrastructure deficits, such as informal dumping, can exacerbate the degradation of urban and peri-urban ecosystems that would otherwise buffer climate risk [9].

Nature-based solutions (NbS), including ecosystem-based adaptation (EbA), are increasingly promoted to help cities respond to the climate crisis at the local level, and many cities are rapidly investing in them alongside conventional engineered infrastructure [6, 10]. Definitions have evolved since NbS gained prominence in global climate and biodiversity policy in the late 2000s [10]. A multilaterally agreed upon definition adopted under UNEA defines NbS as ‘actions to protect, conserve, restore, sustainably use and manage natural or modified terrestrial, freshwater, coastal and marine ecosystems which address social, economic and environmental challenges effectively and adaptively, while simultaneously providing human well-being, ecosystem services, resilience and biodiversity benefits’ [11]. EbA is a more specific approach, defined by the IPCC and used here as ‘the use of ecosystem management activities to increase the resilience and reduce the vulnerability of people and ecosystems to climate change’ [12]. Differences between them are based on scope. NbS serves as an ‘umbrella concept’ that encompasses interventions and actions that address a wide range of societal challenges, including food and water security, human health, disaster risk reduction, environmental degradation, and climate change. EbA falls under the NbS umbrella but is issue-specific, focusing primarily on climate change adaptation [13, 14]. While adaptation is the primary goal of EbA, it has also demonstrated broader co-benefits for mitigation and development, including in Africa [15].

Across the globe, the appeal of NbS, including EbA, for climate action in cities is centred on nature’s contributions to people (NCPs). These are the multiple benefits and detriments that people derive from and co-produce with nature [16], including climate risk reduction and supporting human wellbeing. The concept of NCPs was developed to broaden the earlier ecosystem services framework and has been used in global assessments, including IPBES [16]. In this review, we distinguish between contributions or outcomes that reduce hazard impacts, such as heat, flooding, drought, coastal erosion and storm surges, and outcomes that increase adaptive capacity, such as improved livelihoods, food security, health, wellbeing and access to ecosystem services. Ecosystems can also contribute to mitigation through carbon sequestration and, in some contexts, reduced energy demand [16].

Although accelerating NbS implementation across cities offers significant potential to match the urgency of the climate crisis [6], concerns persist about their utility for cities of the Global South. NbS concepts and approaches have been developed primarily in Global North contexts [8]. Global reviews and mapping studies show that NbS, and particularly EbA research and practice are geographically biased towards the Global North [17]. This reflects either a paucity of studies or a lack of comprehensive approaches to evidence syntheses, especially for certain Global South regions [6, 10, 17]. As a result, global reviews often reproduce conceptual and empirical biases that limit their applicability to the socio‐economic, governance, and environmental contexts of African cities [18].

The uptake of these terms has been uneven across regions and literatures [19, 20]. EbA entered international policy discourse earlier than NbS, with the Convention on Biological Diversity (CBD) defining EbA in 2009 as the use of biodiversity and ecosystem services to help people adapt to the adverse effects of climate change [21]. The IUCN adopted a formal NbS definition in 2016 [22]. In contrast, much African urban scholarship and practice has long discussed related interventions through terms such as ecosystem services, ecological infrastructure, green infrastructure, urban agriculture, community greening, drainage, watershed rehabilitation, or hybrid infrastructure, often without using the NbS or EbA labels. This implies that delayed or divergent terminology should not be read as an absence of practice. Rather, African urban NbS evidence may be partly hidden within older or adjacent literatures on informality, service delivery, urban ecology, livelihoods, and infrastructure heterogeneity.

It is imperative that Global South evidence informs Global South NbS policy and practice [8]. Global South cities offer long-standing hybrid infrastructure landscapes where decentralised alternatives operate alongside or connect with conventional systems [7, 23, 24]. The strong geographical bias in NbS, including EbA, knowledge production and synthesis hampers implementation and wider adoption in policy and practice. For example, in Africa, limited references to NbS in national adaptation plans have been linked to political unwillingness to fund and promote NbS [10]. These gaps mirror broader biases in urban climate research, where evidence gaps are particularly acute for vulnerable cities in Africa [25]. Notably, despite a proliferation of systematic reviews focused on NbS in urban or built‐up environments, none has been dedicated specifically to African urban contexts, leaving a significant gap in regionally grounded assessments of NbS for climate change adaptation [18]. This is particularly important for the social dimensions of NbS, particularly EbA, which also remain insufficiently examined, in relation to justice, equity and distributional trade‐offs in dense urban environments [18]. Without systematic review evidence that attends to these dynamics, there is a risk that NbS interventions designed in and for Global North ecologies, infrastructure and societies may inadvertently cause conflict and reinforce existing vulnerabilities rather than reduce them [8, 26].

Reviews of the urban NbS literature relevant to Africa remain partial and uneven in scope. Existing work includes narrative reviews of green infrastructure in informal settlements in developing countries [27], urban flood mitigation techniques [28], cases of NbS for urban agriculture in Africa [29], a systematic review on NbS for hydro-meteorological risk mitigation in sub-Saharan Africa [30], global systematic reviews focused on specific functions (e.g. urban cooling and energy outcomes [31]), and Global South case study analyses of NbS, including EbA [10]. Global synthesis efforts also include a thematic review of NbS reviews combined with expert assessment to identify barriers and pathways for scaling in cities [6] and global mapping of urban EbA [17].

To strengthen the evidence base for urban climate action in Africa, systematic and reproducible approaches are needed to identify and synthesise the fast-growing NbS literature, including both peer-reviewed and grey literature. This is important because major science-policy assessments, including those of the IPCC, translate dispersed research into decision-relevant knowledge. Yet, without evidence synthesis efforts to pre-aggregate the underlying literature, global assessments such as the IPCC Special Report on Climate Change and Cities risk incomplete and geographically biased NbS coverage, limiting the visibility of urban NbS, including EbA, in Africa and constraining uptake [25]. Moreover, the literature highlights the limited availability of financing as a core barrier to the adoption of NbS, but systematic analysis of financing mechanisms tailored to African urban contexts is still lacking, constraining both policy uptake and pathways for scaling implementation [18].

To improve coverage of African literature and solutions at the science-policy-practice interface and to generate targeted Africa-centric policy advice, we undertake a continental assessment of NbS, including EbA, for climate action in African cities, covering the 55 member states of the African Union and the islands of Mayotte, Réunion and the Canary Islands. To ensure we do not miss smaller cities that could be particularly vulnerable to climate change, we adopt an inclusive definition of cities as continuous built-up areas with a population of at least 10 000 people [32, 33]. We focus on the following core questions: (i) what types of NbS are implemented for climate adaptation and mitigation in African cities? and (ii) what factors shape implementation, effectiveness and scaling? Acknowledging that informality is widespread and growing in African cities, and that challenges and opportunities for climate action in these contexts may differ from formally planned and serviced areas, we also seek to identify what types of NbS are being implemented in informal settlements, and how are these being governed.

To answer these questions, this paper presents a synthesis of the NbS actions being implemented in African cities, followed by a summary of their outcomes, evidence on their effectiveness, and factors influencing their feasibility.

## 2. Methods

### 2.1. Information sources and search strategy

The development of our dataset followed the systematic review protocol outlined by Rule *et al* [34] according to the Preferred Reporting Items for Systematic Reviews and Meta-Analyses (PRISMA) 2020 framework. Peer-reviewed literature was gathered by searching the Title-Abstract-Keyword field in Scopus and the Topic field in the Web of Science Core Collection. Searches were conducted in August 2025 and limited to publications from 2005–2025. The search start date was set to reflect the publication of the Millennium Ecosystem Assessment in 2005 [35] in which the ecosystem services framework was articulated, linking ecosystems to human wellbeing. Search terms are provided in full in table S1 and followed the logic below:

[(African_Cities_List OR ((Africa_term OR African_Countries_List) AND urban_term)) AND (climate_term OR mitigation_term OR adaptation_term) AND ecosystem_term]

The list of African cities used in the search string was derived from the UNDESA list of settlements with a population of more than 300 000 people [36, 37], as the most recent and comprehensive list of African urban areas available. However, this list was not used to exclude studies. Common translations of African city and country names were included in the search strings.

Adaptation terms related to common climate hazards, such as heat, floods, and drought. Ecosystem terms included EbA, NbS and other terms relating urban ecosystems to climate functions that are commonly used in research and practice, like ecosystem services. This enabled us to identify relevant studies even when they did not employ the NbS or EbA framing. This was particularly important for the African urban literature, where implemented practices may pre-date or sit outside the formal adoption of NbS/EbA terminology. We therefore treated NbS and EbA as analytical framings applied to implemented ecosystem-based actions that met our inclusion criteria, rather than as keywords that studies themselves were required to use. To constrain our search string within database limits, we did not include terms relating to specific NbS actions (e.g. tree planting, desilting). It is possible, therefore, that our searches were not exhaustive of relevant studies. However, the search strings were iteratively tested and refined for sensitivity and precision.

To incorporate knowledge beyond the confines of peer-reviewed research, we identified relevant sources of grey literature based on consultation with practitioners working on NbS in African cities and searched curated databases and targeted organisational repositories (listed in table S2). These repositories were searched or filtered as their functionality allowed for resources relevant to Africa, cities, climate change responses, and/or ecosystems, and included document types. Where search or filter functions were not available or not effective, resource lists were manually pre-screen based on titles and metadata (table S2). We did not use Google Scholar to search for additional resources, as the maximum search string length was too short to incorporate the main topic areas of our search string. Only digitally archived grey literature was included in our screening process, and it is possible that other relevant reports were missed due to the limited digital archiving of reports on NbS implementation in African cities.

In addition to these searches, we harvested references from review articles in the field and from grey literature reports synthesising evidence on NbS, including EbA. Search results were managed using Zotero reference manager and Covidence software. After duplicates were removed, 1765 studies were incorporated into the screening process (figure 1).

**Figure 1.**
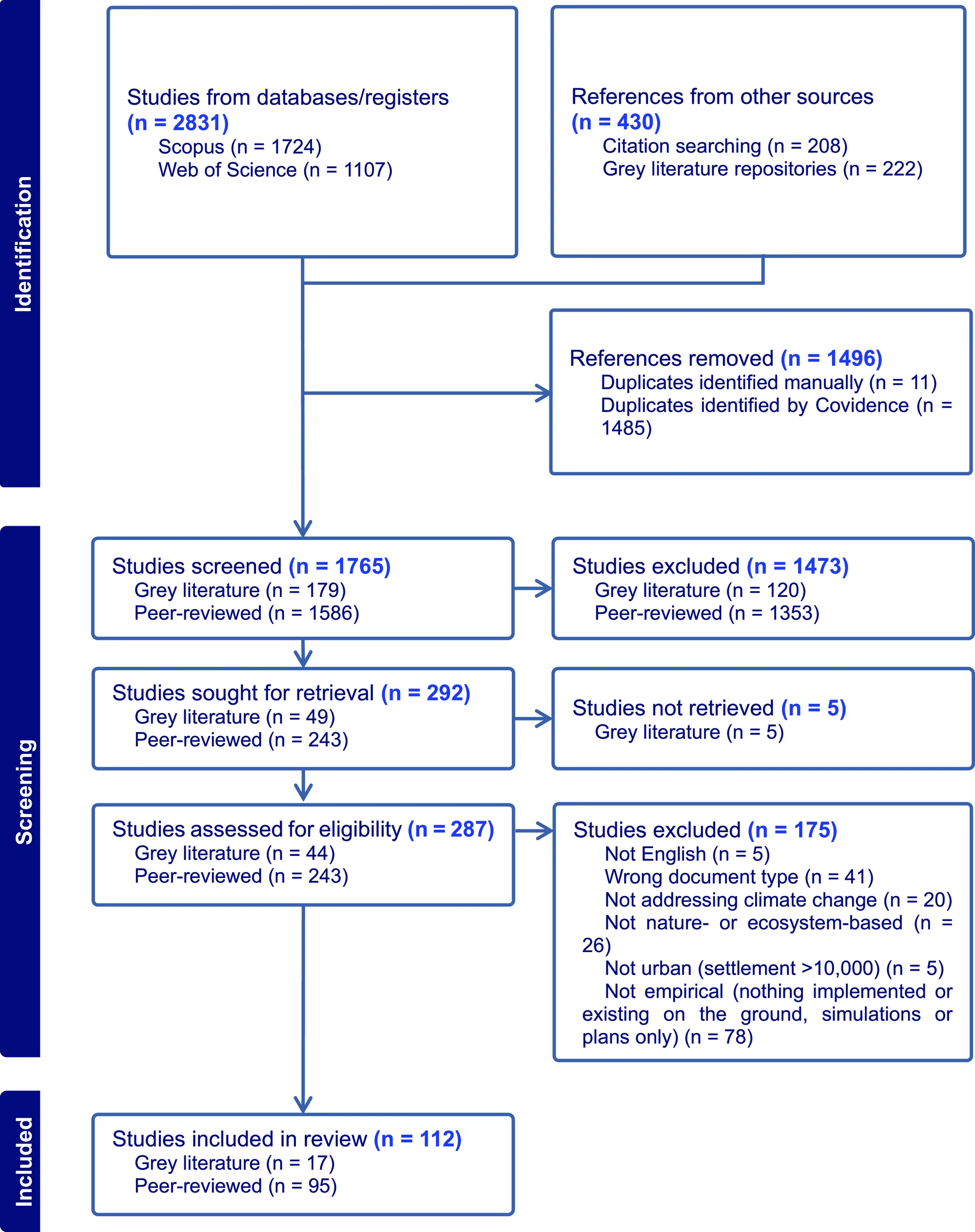
PRISMA 2020 diagram reporting literature identification and screening for a systematic review of implemented NbS in African cities.

### 2.2. Eligibility criteria and screening

Two rounds of screening were undertaken using the Covidence platform: title and abstract screening, and full text review. For both rounds, all studies were screened by two reviewers blindly, and screening decisions were compared. Conflicts were resolved by a third reviewer or by discussion among the reviewer team. Of the 1765 studies screened, 1473 were excluded after title and abstract screening. Full texts were retrieved through the Cape Town and Utrecht University libraries. Five grey literature reports were not publicly available or could not be shared by their authors for full text review. Full text review was conducted for 287 studies, of which 175 studies were excluded based on the criteria in table 1. After full text screening, 112 studies were included in the final dataset. This process is reported using the PRISMA framework in figure 1.

**Table 1.** Inclusion and exclusion criteria for the screening of peer-reviewed and grey literature for inclusion in the systematic review.

Inclusion	Exclusion
• Research articles, book chapters, project reports and grey literature reporting empirical quantitative, qualitative or mixed methods data. • Studies focusing on at least one African urban settlement with a population greater than 10 000 people. • Projects or programmes where implementation is ongoing or complete. • Studies of urban ecosystem creation, restoration or existing ecosystems or urban green or blue spaces that are sustainably managed or protected. • Studies providing evidence of urban ecosystems or natural spaces that reduce the incidence or impacts of climate hazards, increase resilience and adaptive capacity, reduce greenhouse gas emissions or sequester carbon, or are managed to provide at least one of these benefits. • Studies and reports written in English.	• Reviews, opinion pieces, editorials, commentaries, and conference papers without clear empirical evidence base. • Conceptual papers or grey literature reports with no primary empirical evidence reported. • Projections, modelling or prospective studies, proposals or plans where no NbS actions have been implemented. • Studies on human settlements with a population less than 10 000 people. • Studies considering only rural settlements, agricultural ecosystems or protected areas outside of urban areas. • Studies without evidence on African urban ecosystems. • Studies on ecosystems or natural spaces where the impacts on climate hazards, resilience and adaptive capacity, greenhouse gas emissions, or carbon sequestration are not considered. • Studies and reports published before 2005. • Studies or reports of climate responses not involving nature or ecosystems. • Studies or reports in other languages.

The studies included in this systematic review report primary empirical evidence based on quantitative, qualitative and mixed-methods data of existing or implemented NbS including EbA in African cities. Studies and reports of planned or modelled NbS that had not yet been implemented were excluded, as were reviews, commentaries or conceptual reports that did not provide primary empirical evidence. The same inclusion criteria were applied to peer reviewed and grey literature (table 1).

### 2.3. Data extraction and coding

Data from all included studies were extracted and coded independently by two reviewers in Covidence. The extraction template is provided in table S3 and included: study metadata; location and settlement type; research methods; NbS description, intervention type and ecosystem type; stakeholder involvement, governance and funding; NbS outcomes, in terms of climate benefits, co-benefits, disservices and trade-offs; factors affecting outcomes; inclusion of equity considerations [34]. The data extraction and coding process combined deductive categories based on existing NbS typologies and reviews [38–41], including for barriers and enablers (table S4) with inductive development of themes and qualitative analysis from our dataset.

To facilitate an inclusive and cross-disciplinary review, no studies were excluded on the basis of quality appraisal. Grey literature reports, and peer-reviewed studies across different disciplines follow different standards of reporting and transparency and the use of quality appraisal criteria in screening may have resulted in the exclusion of valuable grey and other literature from our corpus. Instead, a set of transparency and validity indicators was included in the data extraction and coding process (table S5) and used to weight confidence in the results. To maintain procedural independence, reviewers were not involved in screening or extracting data from studies of which they were an author or where they had been involved in implementing the initiative described.

### 2.4. Synthesis approach

The synthesis and presentation of results is split into four sections. The Actions and Outcomes sections provide a synthesis of the NbS actions implemented in African cities and how these contribute to climate change adaptation and mitigation. The Effectiveness and Feasibility sections present a synthesis of the factors influencing the implementation, outcomes and scaling of NbS initiatives. The following sections detail the analysis approach for each of these areas.

#### 2.4.1. Actions and typology derivation

To synthesise the NbS actions being implemented in African cities, we developed a typology for NbS actions. A typology is a system of classification, used here, to provide an accessible and grounded synthesis of implemented urban NbS actions in Africa, rather than to propose a new, universally applicable classification. Each class in our typology is associated with a particular unit or units of intervention for NbS actions (e.g. trees, parks, rivers, food gardens). We organised NbS actions by unit of intervention to ground the typology in actions implemented in the urban African context. The typology classes were developed through an iterative, inductive process. This involved the production of a summary of NbS actions from across the corpus based on the extracted data. The author team iteratively developed and refined the typology classes, moving between the classes and individual studies to refine class boundaries and clarify units of intervention. The typology classes are: T1—Green spaces, T2—Rivers, catchments and drainage systems, T3—Coastal systems, T4—Green buildings, and T5—Urban agriculture and multi-use gardens.

After developing the typology classes, each study (*n* = 112) was classified into the typology at three levels. First, for each study, one primary class was coded, based on the implemented actions and goals of the NbS initiative and study. This classification was done to capture the main focus of each study and to avoid double-counting. However, many studies in the corpus span multiple actions and units of intervention, for example, river rehabilitation projects with pocket parks and bioswales, or peri-urban catchment work including agroforestry and vegetating riparian zones. Classifying a study into a single class can under-represent important secondary modes. For this reason, a secondary classification or multi-class count, was done, including multiple classes per study where relevant. Finally, for studies including multiple cities (*n* = 26), a per city multi-class classification was done to facilitate mapping of implemented NbS actions across African cities. In studies where similar actions were implemented across cities (*n* = 16), all cities were classified into the same typology classes. Where actions differed across cities (*n* = 10), classes were assigned to each city occurring in that study.

This classification was done by two authors independently, coding the action description, ecosystems and spaces of implementation, NbS mode and study aims. Where actions in one study fell across multiple classes, the primary class was coded according to the largest number or reach of actions, and/or the main aims of the NbS intervention. Differences in classification were resolved through discussion. There was an agreement rate of more than 85% between the two coders for both primary and multi-class coding.

GIS mapping was undertaken at continental and sub-continental scales to indicate the geographical coverage of included studies and spatial congruence with global biodiversity hotspots [42]. Where studies had an explicit focus on and/or named informal settlements in which actions were implemented, these were coded and used to synthesise NbS actions in informal settlements.

#### 2.4.2. Outcomes of NbS implementation

Outcomes of NbS initiatives were coded through a combination of deductive and inductive approaches, based on the NCPs framework and its links to climate action presented in the Introduction [16]. We coded outcomes reported by studies based on qualitative and quantitative data. We did not apply thresholds for the magnitude of outcomes or meta-analytic strategies, as the scale of NbS interventions varied widely from individual households to multi-city programmes. Climate change mitigation outcomes combine carbon sequestration and reduction of greenhouse gas emissions. Climate change adaptation outcomes are analysed in two categories: reducing hazard impacts and increasing adaptive capacity. Reducing hazard impacts includes outcomes that directly addressed particular climate hazards, for example, heat mitigation, flood risk reduction, and reduced coastal erosion. We coded livelihood and employment outcomes as increased adaptive capacity only where the study explicitly linked these outcomes to reduced vulnerability, improved coping or recovery capacity, reduced sensitivity to climate hazards, or enhanced livelihood security in climate-exposed populations. Livelihood or employment benefits that were reported without such a climate-risk linkage were coded as social benefits, but not as adaptation outcomes.

We also analysed outcomes in terms of social and biodiversity benefits. Like the climate action outcomes, this analysis was undertaken by coding outcomes reported by studies based on quantitative and qualitative data. Social benefits coded included increased employment, strengthened livelihood opportunities, improved health and wellbeing, increased safety and security, increased recreational opportunities, among others. Coding for climate change adaptation outcomes overlapped with coding for social benefits where strengthened livelihoods and employment opportunities were reported as reducing vulnerability to climate change. This distinction reduces the risk of inflating adaptation claims while recognising that, in highly exposed and resource-constrained urban contexts, livelihood security can be a central pathway through which NbS reduces climate vulnerability. In addition to climate change adaptation, mitigation, and social outcomes, we also present the number of included studies that report outcomes for the protection or improvement of biodiversity through the NbS interventions. Our coding of biodiversity outcomes did not require a link to be made between biodiversity and vulnerability to climate change.

#### 2.4.3. Evidence of effectiveness

As with Outcomes, the analysis of the effectiveness of NbS interventions was based on a narrative overview of self-reported evidence of effects in included studies rather than on a quantitative meta-analysis of climate adaptation or mitigation results. This approach was taken to be inclusive of evidence from across disciplines, scales and sectors and the reporting of both qualitative and quantitative data. To analyse effectiveness, the aims and results in terms of climate change adaptation and mitigation were coded for each study and grouped by climate hazard or mitigation impact. For studies that include this information, we coded and report the type of evidence presented, the magnitude of outcomes, and the modifiers of effectiveness in the narrative overview.

#### 2.4.4. Factors influencing NbS feasibility

The feasibility of NbS interventions was assessed across several thematic areas. Combined inductive and deductive coding was used to synthesise the most frequently reported barriers and enablers to the implementation and scaling of NbS reported across the corpus. Barriers and enablers were coded against predefined categories in the extraction template (tables S3 and S4), drawn from the NbS literature used to develop the coding framework (section 2.3), with free-text fields (questions 7.2 and 7.4) to record additional factors. A category was coded only where a study reported that factor as hindering, enabling or contributing to the effectiveness of the implemented initiative. Section 3.5 presents the results of this analysis. Stakeholders involved in NbS interventions were identified from each study across multiple stages (planning, implementation, maintenance), alongside beneficiary groups and stakeholder groups contributing to funding NbS activities. These were synthesised through a narrative approach across the corpus and analysed alongside governance and cost-related barriers and enablers. Trade-offs and unintended outcomes of NbS initiatives were also coded and grouped thematically for narrative synthesis.

## 3. Results

Five key messages emerge from the Results and are elaborated in the sections that follow. First, NbS, including EbA, are implemented widely across African cities, but the evidence is concentrated in a small number of large cities, with most urban areas represented by a single study (Section 3.2; figure 2). Second, green spaces dominate the evidence, yet many initiatives are hybrid, combining actions across typology classes. Urban agriculture and multi-use gardens are rarely the primary focus of these studies but are embedded in a third of initiatives (Section 3.2). Third, reported climate change outcomes are predominantly for adaptation rather than mitigation. Adaptation is delivered through two often-combined pathways of reducing harmful impacts from climate hazards and strengthening livelihood security. Beyond climate outcomes, more than half of studies (*n* = 65) report biodiversity gains across all five typology classes, and nearly half of the urban areas with implemented NbS lie within global biodiversity hotspots, indicating potential synergies between urban climate action and biodiversity protection (section 3.3; figure 3). Fourth, reported effectiveness is largely positive but rests mainly on qualitative, self-reported and short-term evidence, with heat mitigation the most consistently quantified outcome (section 3.4). Fifth, feasibility is determined more by governance and finance than intervention type. Local governments, residents, and community actors carry much of the work of implementation and maintenance, with community actors more prominent in informal settlements. Inadequate support for long-term maintenance and monitoring was the most frequently reported barrier (section 3.5), reflecting funding concentrations in planning and initial implementation.

**Figure 2.**
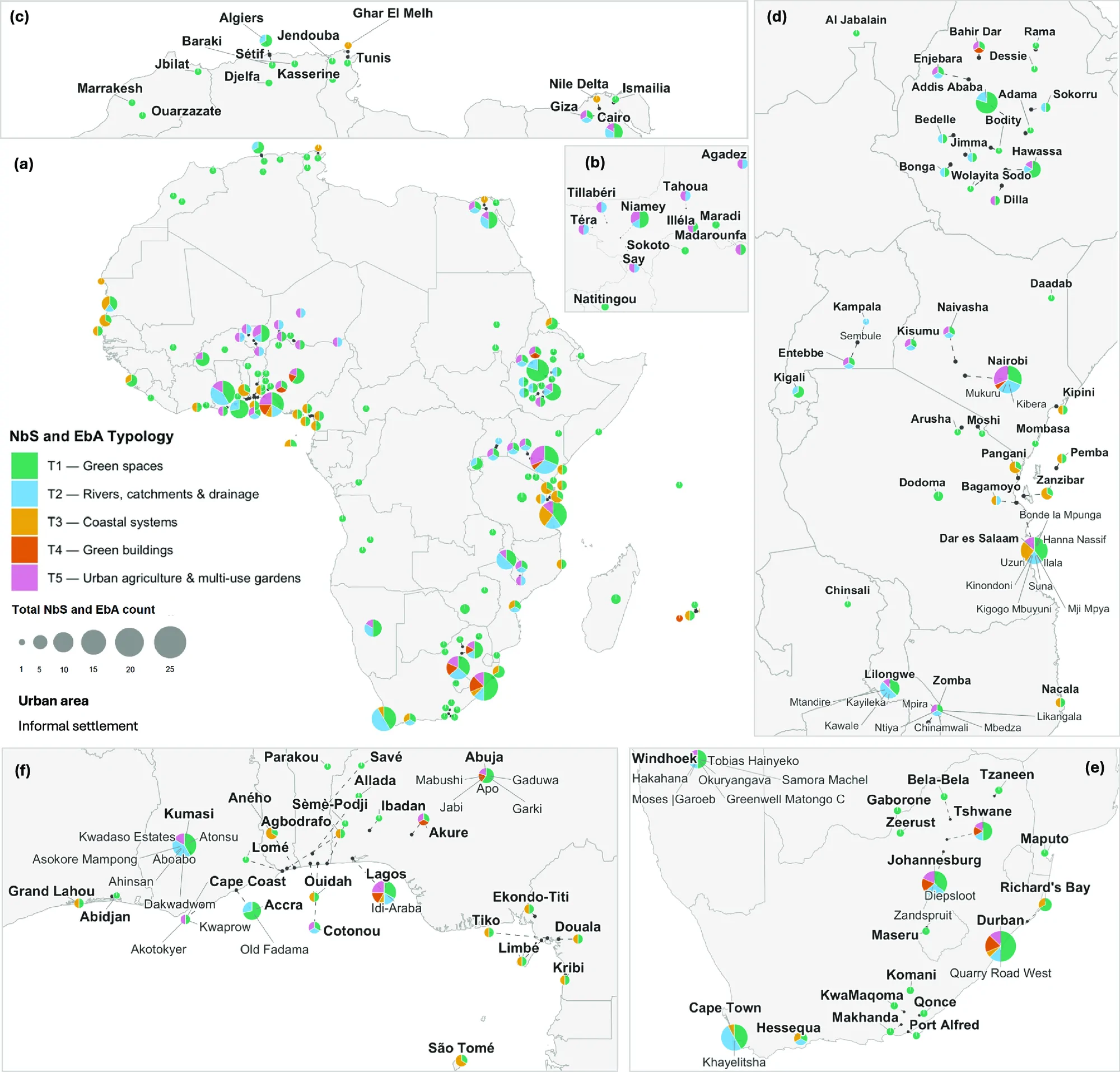
Typological composition of nature-based solutions (NbS) and ecosystem-based adaptation (EbA) reported across African cities. Pie charts show the composition of NbS, including EbA, reported in 134 African cities and named informal settlement areas across 112 studies. Symbol size is scaled to each city’s total location count, defined as the number of study × city × typology-class occurrences. Pie segments show the proportional share of the five typology classes: T1 Green spaces; T2 Rivers, catchments and drainage systems; T3 Coastal systems; T4 Green buildings; and T5 Urban agriculture and multi-use gardens. Named informal settlements, or larger areas containing informal settlements, where NbS were reported are given in light text and linked to their parent urban area. Regional insets disambiguate clustered locations. Because one study may cover several cities and one city-study record may include several typology labels, the total multi-label coverage count exceeds the number of unique studies.

**Figure 3.**
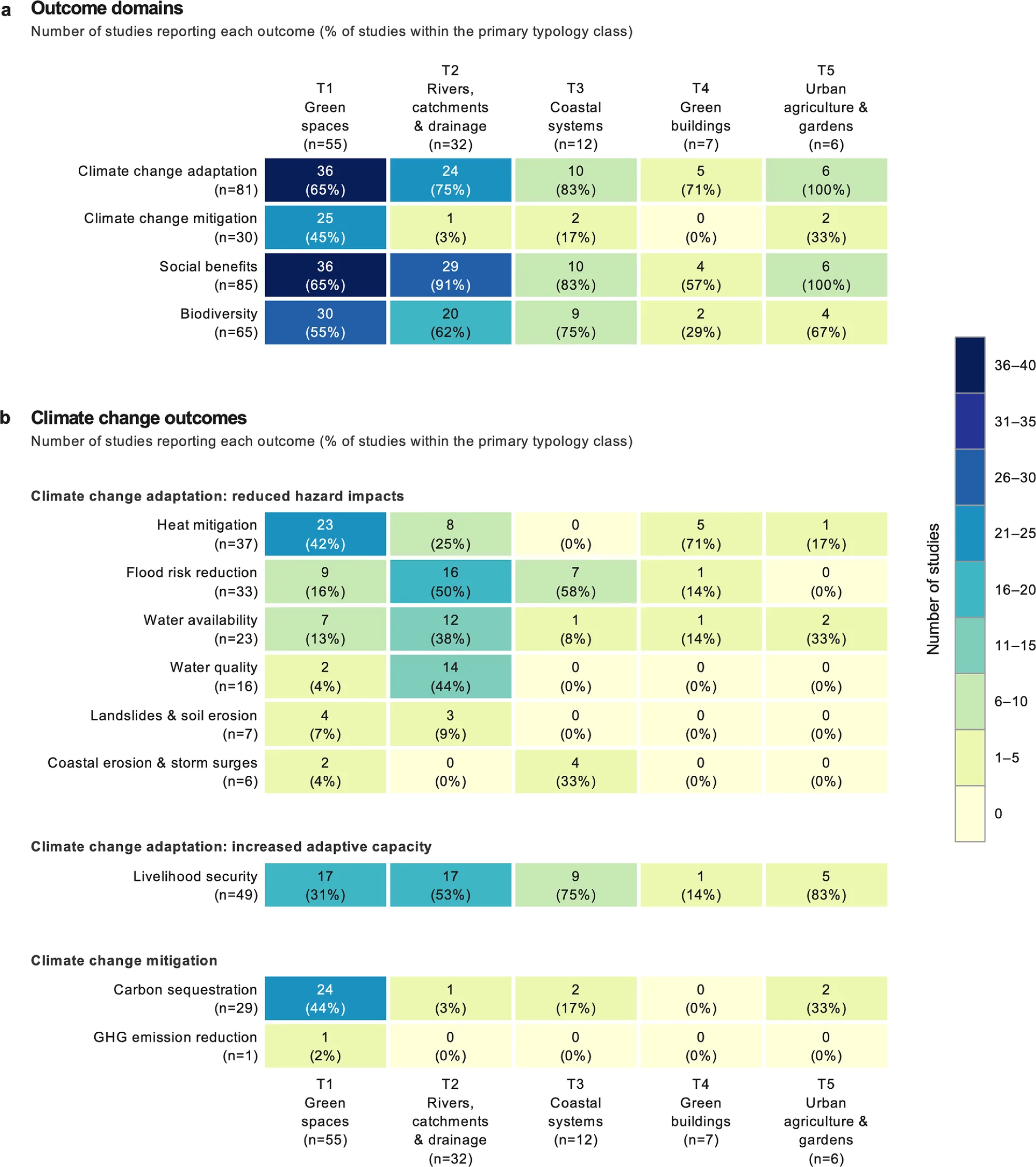
Climate, social and biodiversity outcomes reported for urban nature-based solutions (NbS) by primary typology class. Heatmaps show the number of studies (*n* = 112) reporting outcomes of urban NbS, including ecosystem-based adaptation (EbA), in African cities, grouped by each study’s primary NbS typology class. Panel a shows the four outcome domains: climate change adaptation, climate change mitigation, social benefits, and biodiversity. Panel b disaggregates climate outcomes into reduced hazard impacts, increased adaptive capacity through livelihood security, and mitigation. Values in parentheses give the percentage of studies within each primary typology class that report the outcome; n beside each outcome gives the number of studies reporting it across all classes, and n beneath each class gives the number of studies in that class. Outcomes are counted whether or not they were an explicit aim of the intervention; figure 4 reports success only among studies that targeted the corresponding outcome. Studies can report more than one outcome, so counts are not mutually exclusive down columns. Counts are based on primary typology allocation, so each study contributes to only one typology class. The colour scale represents study counts in bins of five and is fixed from 0 to 40 across both panels.

### 3.1. Overview of evidence

Of the 112 included studies, 17 (15%) are grey literature reports, including project reports, project evaluations, and empirical case studies in thematic reports. The remaining 95 studies are peer reviewed journal articles and book chapters. Although the search strategy incorporated studies from 2005 onwards, the earliest included study was published in 2010, with a steady increase to 2025 (figure S1), reflecting the growing interest in and application of NbS and EbA to African cities. This growing publication trend was likely influenced by the growing global attention to and investment in NbS, including EbA, over this period. For example, the United Nations CBD first defined EbA in 2009 [21], followed in 2016 by an influential definition of NbS from the IUCN [22]. The funding and mainstreaming of urban NbS also grew over this period including through the European Commission’s Horizon 2020 programme, among others.

Most of the included studies focused on a single urban area (*n* = 84), or multiple urban areas within one country (*n* = 11). Of the multi-country studies, nine focused only on African countries, one on the Arab Region (including North Africa) [43], and seven had a cross-continental or global focus. The majority of first authors of the corpus are Africa‐based (62%), with strong representation from Southern, Western and Eastern Africa (most notably South Africa, Ethiopia, Nigeria and Ghana). Non‐African affiliations of first authors are mostly from the Global North countries (29%), reflecting established research hubs that also publish on African urban NbS and EbA such as Germany, the United Kingdom, and the Netherlands. Other regions outside Africa and the Global North account for just 9% of first author affiliations.

### 3.2. Implemented NbS actions in urban Africa

Evidence from across the continent informed the development of the first typology that reflects literature on NbS implementation in African cities (table 2). The typology organises NbS measures, including EbA, into five classes based on their intervention unit (e.g. rivers, parks, buildings) to enable an empirically grounded synthesis. Alongside the units of intervention and actions included in each class, table 2 presents the classification of studies by primary class (what the study is mainly about), multi-class (which intervention classes are included in the study) and location (where each intervention type is being implemented). For example, while only 12 of the 112 studies focused primarily on coastal ecosystems, actions involving Coastal systems were included in 19 studies. In addition, there were 42 instances of Coastal system NbS measures being implemented when accounting for studies with multiple locations (table 2). Together, these counts provide a balanced view of programme composition without relabelling or inflating counts. The following paragraphs describe the range of interventions in each class relative to their scale and mode of NbS intervention (protection, restoration, sustainable management or creation of ecosystems).

**Table 2.** Typology of implemented NbS actions in African cities. Five typology classes are defined by units of intervention, each with a distinct set of actions. Each included study (*n* = 112) was labelled with one primary class, indicating the dominant actions. This primary class count asks ‘What is each study mainly about?’ Where multiple actions in different classes were implemented or actions had multiple units of intervention, studies were labelled with multiple classes, indicating the hybridity of NbS actions. This multi-class count asks, ‘Which intervention types does each study include?’ Classes were also labelled per location for studies covering multiple urban areas, indicating the geographic coverage of intervention types. This location count interrogates from the corpus ‘Where does each intervention type show up on the ground?.’ For example, a study spanning two cities and marking three typologies in each contributes six counts in total.

Typology class	Unit(s) of intervention	Description of actions	Primary class count (# studies)	Multi-class count (# studies)	Location count (# study-city tags)	Example studies
**T1 Green spaces**	Urban green spaces, parks, gardens, trees, natural to semi-natural vegetated areas	• Creating, upgrading and managing parks and public open spaces, including forests; • Restoring and stewarding urban natural mixed vegetation areas including invasive alien plant clearing as part of green-space management; • Planting, growing, conserving and monitoring trees including street trees and trees at institutional sites (e.g. schools, churches); • Mass campaigns for city-wide tree planting; • Reforesting or restoring degraded urban land; • Designating and managing inland protected-area networks; • Area-based legal and planning protection of inland vegetated ecosystems.	55	92	190	[44–53]
**T2 Rivers, catchments and drainage systems**	Rivers, streams, inland wetlands, aquifers, catchments, watersheds, bio-retention areas, green-grey-blue drainage systems	• Managing the urban water cycle through nature at regional, local and source scales; • Restoring and protecting natural inland wetlands; • Rehabilitating and naturalising rivers and streams; • Planting and restoring riparian ecosystems; • Sustainably managing watersheds and catchments; • Creating sustainable urban drainage (SuDS) or hybrid grey-green-blue infrastructure including bioswales, infiltration trenches, soakaways, permeable paving, stormwater ponds, detention and retention basins; • Constructing wetlands for water treatment and retention; • Rainwater harvesting at site or catchment scale. Green-blue actions are classified primarily as T2 where the primary purpose is water cycle regulation or water-related ecosystem services.	32	49	66	[8, 9, 54–59]
**T3 Coastal systems**	Coastal wetlands, estuaries, coastal dunes, intertidal zones, coral reefs, near-shore marine ecosystems	• Restoring and stabilising coastal ecosystems to protect against sea-level rise, storm surges and erosion, and saline intrusion; • Restoring, conserving and managing mangrove forests; • Restoring and stabilising coastal dunes, including through sand fences, and vegetation belts; • Beach and sand nourishment; • Rehabilitating coral reefs; • Estuarine, salt-marsh and seagrass interventions, including living shorelines; • Sustainably managing coastal fisheries and marine resource harvesting; • Protecting coastal and near-shore ecosystems. Actions in lagoons and deltas are classified as primarily in T3 where the primary purpose is addressing sea-level rise, coastal erosion or other coastal hazards.	12	19	42	[13, 60, 61, 62–64]
**T4 Green buildings**	Buildings, dwellings	• Incorporating vegetation into building envelopes; • Creating green roofs, living walls and vertical greening systems; • Creating and managing building-integrated agriculture and productive envelopes. Building-integrated food gardens are primarily classified under T4.	7	14	14	[65–68]

**T5 Urban agriculture and multi-use gardens**	Urban and peri-urban farms, food gardens, multi-use gardens	• Producing food and biomass through urban and peri-urban planting; • Planting and maintaining backyard and household food gardens; • Planting and maintaining communal, sack and bag gardens; • Implementing urban and peri-urban agroforestry; • Restoring land for edible or productive uses. Restoration of riparian and other ecosystems on agricultural land is primarily classified as T5 where the primary purpose is to support production.	6	38	47	[69–72]

Urban green spaces (T1) were the most frequently reported NbS class, with 82% of studies (*n*= 92) reporting green space-related actions. These actions span the four NbS modes and multiple scales, from individual parks or backyard gardens to neighbourhoods and city-wide green space networks. The legal and customary protection of urban forests, for example, was reported in Accra (Ghana) [44], and Ethiopia [73–75], among others. Sustainable management of resource harvesting from parks and green spaces was reported in several towns in South Africa [45]. Tree planting campaigns across the continent contributed to both the restoration of forest and woodland ecosystems, and the creation of new gardens and green spaces through design-based NbS [46, 76].

NbS interventions focused on Rivers, catchments, and drainage systems were the next most frequently-reported class, with actions appearing in 49 studies. Like green space interventions, this class spanned multiple scales and NbS modes. Creation-based NbS included the construction of wetlands, bio-retention areas, rainwater harvesting and other hybrid green-blue-grey measures, frequently targeting source- and local-scale stormwater regulation [54, 55, 77, 78]. Sustainable management of catchments and watersheds was often accompanied by restoration through revegetation of riparian corridors and water towers [79, 80]. Other restoration activities in this class included re-naturalising urban streams and restoring degraded wetlands, often at the scale of individual parks or river reaches [56, 81]. Legal protection of wetlands and catchments was reported in South African cities [47, 78, 82], as well as in Nairobi (Kenya) [79].

Coastal system NbS interventions ranged from individual neighbourhoods [60] to multi-country programmes [61]. Restoration was the mostly widely implemented mode, including mangrove reforestation, dune rehabilitation, and coral and seagrass restoration [61, 62, 83]. Sustainable management of coastal fisheries and legal protection accompanied ecosystem restoration in some cases [63]. Creation or design-based NbS in this category included planting on dunes and salt flats [84].

Green building interventions reported were less wide-ranging in terms of scale and NbS modes, focusing on ecosystem creation and sustainable management, typically for a single building or group of buildings. These studies reported on the planting of green roofs [65, 66], and green walls, including vertical gardens and building-integrated agriculture [67, 85, 86]. Sites varied from large commercial and institutional buildings [81] to small low-income dwellings [86].

Finally, urban agriculture and multi-use gardens were the primary focus of only six studies but were incorporated into the actions of 34% (*n* = 38). This reflects the incorporation of food and fibre production into a range of interventions across all primary classes. Urban agriculture and multi-use garden interventions covered a range of scales from peri-urban farmland [69, 70] to backyard food gardens [71]. Intervention modes included the creation of multi-use gardens on degraded urban land [87], the restoration of urban and peri-urban agricultural land to reduce soil erosion [70], and the sustainable management of livestock, agroforestry and other productive systems. Sustainable management of production systems included experimentation and implementation of strategies like bag gardening, mulching, manure management, and agroforestry to reduce water use, run-off and fertiliser inputs [69, 72].

#### 3.2.1. Geographical distribution of NbS interventions

The 112 studies included in this review report on NbS initiatives, including EbA, across 134 urban areas and 41 African countries, as well as Réunion Island. While evidence is concentrated in a few large metropolitan areas, the broad representation indicates the widespread implementation of NbS, including EbA, across the continent.

At the country level, the distribution of implemented NbS varies both by volume of evidence and typology (see figure S2). Reported implementation of NbS typologies (by location count) is clustered in South Africa (*n* = 59), Ethiopia (*n* = 37), Tanzania (*n*= 29), Kenya (*n* = 27), Niger (*n* = 23), Nigeria (*n* = 22), and Ghana (*n* = 21). Within this group, South Africa exhibits strength across multiple typology classes, including Green spaces (*n* = 31), Rivers, catchments and drainage systems (*n* = 13), and Green buildings (*n* = 6). Ethiopia combines Green spaces (*n* = 24) and Rivers, catchments and drainage systems (*n* = 8) with Urban agriculture and multi-use gardens (*n* = 4). The strongest Coastal system portfolios are seen in Tanzania (*n* = 10) and Cameroon (*n* = 5), with Urban agriculture and multi-use gardens being most evident in Niger (*n* = 10) and Kenya (*n* = 7).

Across the African urban sample, NbS initiatives, including EbA, cluster strongly in a set of large cities (figure 2). However, one of the most striking features of the evidence base is its long tail, where 100 urban areas appear in only a single study. This points to a rich but largely overlooked body of practice distributed across secondary cities, mid-sized towns and smaller urban centres from Nacala (Mozambique) to Nouadhibou (Mauritania). These urban areas rarely enter global NbS or EbA discourses, yet they face acute climate vulnerabilities and are experimenting with locally-grounded green infrastructure, urban forestry and coastal ecosystem management in ways the field has yet to fully account for.

Equally significant is the emergence of evidence from North Africa, where a cluster of 13 studies covers Egypt, Algeria, Tunisia, Morocco and Mauritania. Historically absent from African NbS literature, this North African evidence, ranging from coastal restoration and protection initiatives in the Nile Delta (Egypt) [43] and Nouakchott (Mauritania) [84, 88] to urban green space initiatives in Cairo (Egypt) [89] and Algiers (Algeria) [90], substantially extends both the geographic and ecological scope of what is known about NbS, and particularly EbA, in African urban contexts. This signals that the evidence frontier is expanding well beyond the East and Southern African cities that have historically anchored the field.

The urban centres with the strongest evidence include Durban (South Africa), Nairobi (Kenya), Dar es Salaam (Tanzania), Cape Town (South Africa), Lagos (Nigeria), Kumasi (Ghana), Johannesburg (South Africa), Addis Ababa (Ethiopia), Lilongwe (Malawi) and Accra (Ghana). These hubs have the highest number of typology-tagged mentions in our corpus (figure 2). They consistently show activity across multiple typology classes, with especially dense portfolios of Green spaces and Rivers, catchments and drainage systems, reflecting the predominance of parks, street trees, riparian corridors, and sustainable urban drainage (SuDS) programmes in large cities. Coastal system interventions in cities like Dar es Salaam and Cape Town appear alongside strong contributions from Rivers, catchments and drainage systems, consistent with cities where flood exposure, estuarine systems, and peri-urban catchment management must be addressed together.

In West Africa, Lagos, Accra, and Kumasi are prominent, with smaller coverage of many other cities. Here, portfolios blend Green spaces and Green building measures with targeted river and drainage actions. Across inland East Africa, Lilongwe, Nairobi and Addis Ababa show that multi-ecosystem strategies are not restricted to coastal settings. Southern African cities (e.g. Durban, Johannesburg, and Tshwane) stand out for relatively balanced typology mixes.

#### 3.2.2. NbS implementation in informal settlements

While many of the reviewed studies focus on the city level or do not make clear distinctions between formal and informal areas, the corpus includes 45 named informal settlements or areas where informality is present across 15 cities. Twenty studies focus explicitly on climate action in informal settlement areas. Most of these studies (*n* = 13) had a primary focus on Rivers, catchments and drainage systems, addressing common challenges relating to flood risk and pollution. These studies included the creation of source and local-scale SuDS, for example, in Kibera, Nairobi [54, 58] and Diepsloot, Johannesburg [55], as well as restoring river ecosystems in Zomba (Malawi) [26] and Durban [57]. Fewer studies focused primarily on Green spaces (T1, *n* = 4) and Urban agriculture and multi-use gardens (T5, *n* = 2). However, activities in these classes, such as food gardens, tree planting and green space management, were present in most of this subset of studies (T1: *n* = 15, T5: *n* = 11, for multi-class count). As this suggests, many of the NbS implemented for climate action in informal settlements are multifunctional, addressing multiple climate hazards and/or providing social and biodiversity benefits. In Kibera, for example, small green spaces have been created alongside green walls and green roofs, for cooling, windbreaks, to grow vegetables and to provide recreational space [58].

### 3.3. Outcomes of NbS interventions

#### 3.3.1. Outcomes for climate action

Figure 3 shows the number of studies reporting each outcome domain and climate outcome, by primary typology class. At least one climate outcome either for adaptation or mitigation, is reported by 83% of studies across the corpus (*n* = 93). Evidence of successful climate change adaptation outcomes was reported in 81 of the 112 included studies (72%). The evidence of climate change adaptation presented included both outcomes that reduced the impact of specific hazards and evidence of reduced climate vulnerability or an increase in adaptive capacity of urban residents. In terms of hazard reduction outcomes, heat mitigation was the most frequently reported overall (*n* = 37) and for studies focusing on Green spaces (*n* = 23) and Green buildings (*n* = 5). Reduced flood risk was reported as an outcome by 33 studies, particularly those focused on Rivers, catchments and drainage systems (*n* = 16) and Coastal systems (*n* = 7). Improved water availability (*n* = 23), and improved water quality (*n* = 16) were the next most common outcomes, predictably concentrated in studies focused on Rivers, catchments and drainage systems. Fewer studies reported successful outcomes in reducing coastal erosion and storm surges (*n*= 6) and reducing landslides or soil erosion (*n* = 7). Adaptation through improving livelihood security for urban residents was reported by 49 studies, of which 35 combined this adaptation outcome with directly reducing the impacts of one or more climate hazards.

Evidence of achieving climate change mitigation outcomes was reported in 30 studies. Only one study [91] reported the reduction of greenhouse gas emissions resulting from NbS implementation, although this was not quantified. In the remaining 29 studies, NbS contributed to mitigation outcomes through carbon sequestration. Of these 30 studies, 25 focused primarily on urban Green spaces, while the remaining studies on Rivers, catchments and drainage systems (*n* = 1), Coastal systems (*n* = 2), and Urban agriculture and multi-use gardens (*n* = 2) all incorporated elements of urban greening, including tree planting and revegetating degraded ecosystems. Most studies reporting climate mitigation outcomes also reported climate change adaptation outcomes (*n* = 18), indicating that an exclusive focus on climate change mitigation was uncommon among urban NbS studies on the continent.

The NbS interventions reported on in this corpus range in scale from backyard gardens to multi-country programmes. To accommodate this variety, the synthesis of outcomes and effectiveness relies on the quantitative and qualitative reporting of these outcomes in each study, rather than on meta-analysis. Further research and synthesis efforts would be needed to quantify and assess the geographical scale of NbS outcomes in African cities.

#### 3.3.2. Social outcomes

Direct social benefits from NbS are reported in 85 of the 112 studies. These benefits include strengthened livelihoods, improved employment opportunities, increased health and wellbeing, improved food security, recreational opportunities, and improved safety and security, among others. For example, access to urban green space is associated with a range of direct health and wellbeing benefits for urban residents, including improved mental health, opportunities for physical activity and recreation, and thermal comfort regulation. Evidence from small and medium-sized towns across South Africa shows that urban green infrastructure provides critical provisioning and cultural ecosystem services, including recreation, spiritual value, and stress reduction, which are particularly important for low-income households with few alternatives [45]. In Dar es Salaam, dramatic declines in urban green structure due to both climate and non-climatic pressures are observed to degrade the health and wellbeing of informal settlement residents, with cultural ecosystem services ranked as the most highly valued [50]. Temperature regulation of urban green-blue infrastructure is among the best-evidenced health-relevant outcomes in the corpus. In Ibadan, for example, urban green spaces are found to directly improve outdoor thermal comfort and support wellbeing for residents unable to afford active cooling [92]. Despite this evidence on temperature reduction, quantification of heat-related health or mortality impacts from urban cooling remains largely absent from the literature on implemented NbS, including EbA, in African cities, representing a significant evidence gap.

NbS interventions, including EbA, generate employment and livelihood benefits across multiple implementation phases and across a range of skill levels from manual labour through to semi-skilled management and technical monitoring [93]. Across the initiatives that identified target beneficiaries (*n* = 71), low-income communities (*n* = 35) were most frequently mentioned, followed by city-wide populations (*n* = 25), with comparatively fewer initiatives targeting mixed-income groups (*n* = 11). Youth (*n* = 35) and women (*n* = 30) were frequently explicitly included, with participation largely focused on project implementation and maintenance through ‘green jobs,’ training and education programmes, and stewardship. In Freetown (Sierra Leone), the Treetown campaign created more than 1000 jobs across the tree value chain with employment and income benefits valued at US$8.23–10.76 million in a cost-benefit analysis, and individual tree survival was verified through digital tracking [46]. Urban ecosystem restoration in Durban similarly targeted marginalised groups: the Buffelsdraai Landfill Site Buffer Zone programme employed women, young people, veterans and people with disabilities to manage invasive alien plants and restore indigenous vegetation [94]. Where income generation from NbS initiatives has been quantified, returns are modest. For example, vertical greening system prototypes in Agege, Lagos, generated approximately 16 crop rotations per year with some documented revenue from edible and medicinal plant sales, though commercialisation at scale remained constrained by landlord acceptance and capital costs [86]. Urban agriculture and multi-use garden NbS generate livelihood value that extends beyond cash income through reduced household food expenditure, improved nutrition, and reduced vulnerability to food price shocks. This is documented for multifunctional landscape projects in Ghana [87], kitchen garden initiatives in Kibera (Kenya) [58], and tree-based food security projects in South Africa [95].

#### 3.3.3. Outcomes for biodiversity

More than half (*n* = 65) of the studies in this corpus report improvements in biodiversity as an outcome of the studied NbS intervention. Of the African urban areas included in the reviewed studies, 48% are located within global biodiversity hotspots, indicating potential synergies between NbS for climate action and protecting threatened biodiversity (figure S3). Biodiverse cities, such as Cape Town and Durban, have a long history of proactive protection, management and restoration of ecosystems, aligned with climate action strategies, evidenced by mainstreaming within local government [47, 78, 82]. Clear examples of habitat-focused biodiversity outcomes are found in coastal and wetland studies [62, 78], with 64% of coastal system NbS initiatives being implemented in biodiversity hotspots. Here the evidence suggests that NbS implemented on the African continent is playing a role in securing threatened areas of critical biodiversity within coastal areas (figure S3). For example, mangrove ecosystems in Cameroon are documented as supporting 63 species and functioning as fish nurseries for coastal communities [63], while mangrove restoration along the Eritrean and Mauritanian coasts is framed primarily around fisheries productivity and species recovery [84]. In coastal Tanzania, UNEP-funded interventions explicitly link mangrove and seagrass protection to fisheries resource sustainability for dependent communities [62, 83]. Inland wetland restoration in Kisumu (Kenya) documents habitat restoration for fish species in the Lake Victoria basin, with a formal sustainable asset valuation conducted to quantify returns, though specific service provision rates are not reported in this synthesis [70]. Beyond habitat studies, the corpus documents tree species diversity and carbon storage in urban forests [73, 96].

The outcomes reported above describe the range of climate, social and biodiversity benefits documented across the corpus, regardless of whether these benefits were explicit objectives of the intervention. The next section narrows the analysis to evidence of effectiveness: whether studies that targeted a specific climate outcome reported evidence that the intervention contributed to that intended outcome, and what factors appeared to modify that effect.

### 3.4. Evidence of effectiveness of interventions

Targeted climate outcomes differed across the five primary typology classes (figure 4, panels a and c). Green space interventions were most frequently designed to address heat (*n* = 24), followed by carbon sequestration (*n* = 18) and flooding (*n* = 15). NbS interventions in Rivers, catchments or drainage systems sought to address flooding (*n* = 23) and drought (*n* = 15). Consistent with their coastal location and ecological function, Coastal system interventions targeted flooding (*n* = 9), coastal erosion and storm surges (*n* = 7). Green building interventions were implemented primarily to reduce heat (*n* = 5), while Urban agriculture and multi-use gardens had a mix of aims, including addressing droughts (*n* = 3) and flooding (*n* = 2).

**Figure 4.**
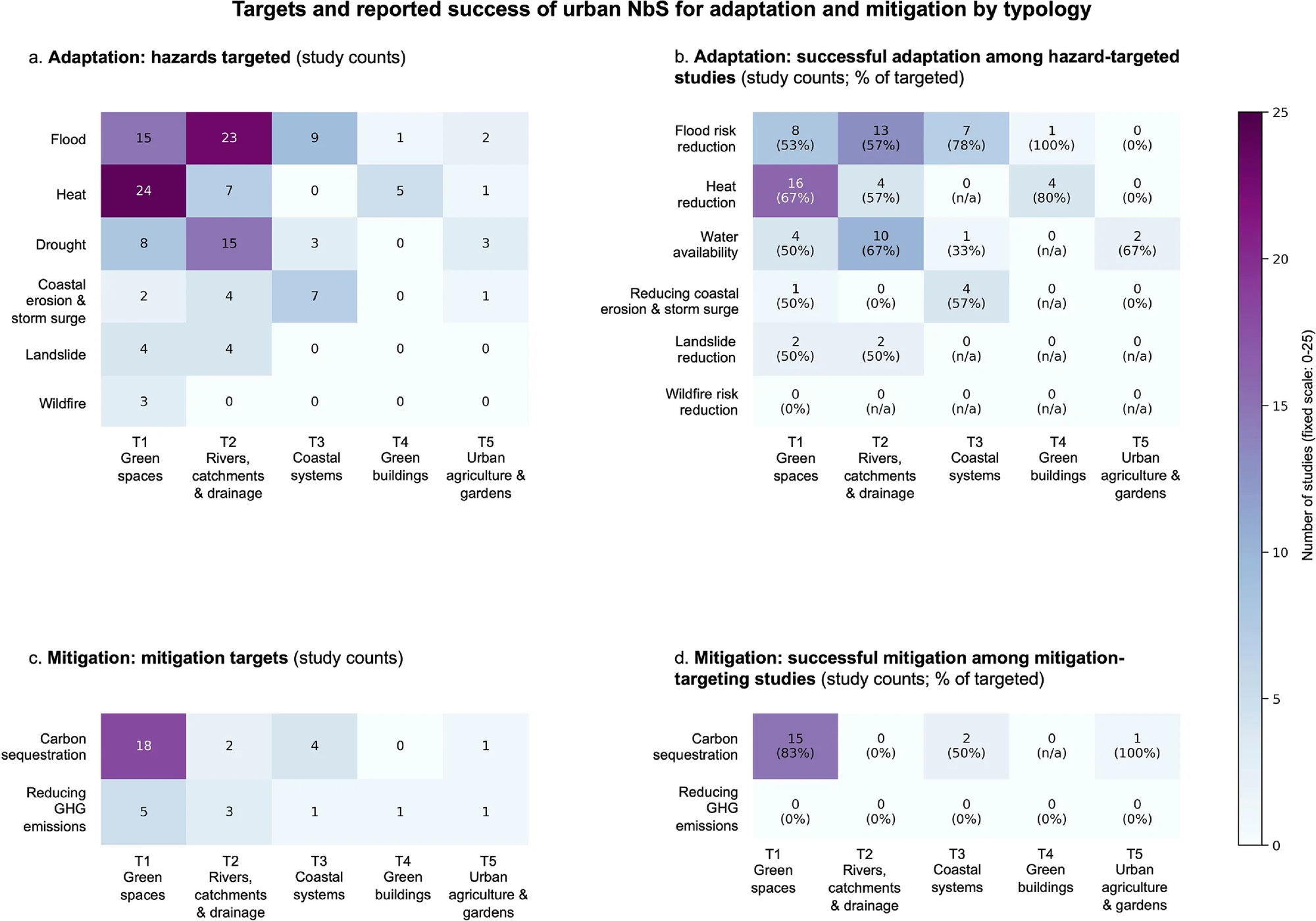
Climate adaptation and mitigation targets and reported success of urban nature-based solutions (NbS) by primary typology. Heatmaps show the number of studies (*n* = 112) reporting climate-related targets and successful outcomes for urban NbS in African cities, grouped by each study’s primary NbS typology class. Panel a shows climate hazards targeted, including flooding, heat, drought, coastal erosion and storm surges, landslides, and wildfires. Panel b shows reported successful adaptation outcomes among studies that targeted the corresponding hazard; values in parentheses indicate the percentage of targeted studies within the same primary typology that reported success. Panel c shows mitigation targets, including carbon sequestration and reducing greenhouse gas emissions. Panel d shows reported successful mitigation outcomes among studies that targeted the corresponding mitigation outcome, with percentages calculated in the same way. Counts are based on primary typology allocation, so each study contributes to only one typology class. The colour scale represents study counts and is fixed from 0 to 25 across all four panels to support comparison between adaptation and mitigation outcomes.

Alongside the targeted hazards and mitigation mechanisms, figure 4 shows the number and proportion of studies that provide evidence of reported successful adaptation (panel (b)) and mitigation (panel (d)) outcomes. These effectiveness counts include only studies that targeted the specific hazards or mitigation mechanisms, in contrast to section 3.3, which includes all studies reporting outcomes, even for hazards that they did not explicitly target. To accommodate the range of scales and evaluation methods in the corpus, we present studies’ self-reported outcomes rather than conducting a meta-analysis based on common indicators.

Heat mitigation is the most consistently quantified adaptation outcome in the corpus, reported on through a range of indicators (summarised in table S6). Local cooling of up to 12.2 °C was reported around parks, green-blue spaces and urban forests. While cooling was consistently reported across the continent from Bulawayo (Zimbabwe) to Algiers (Algeria) [74, 89, 90, 97, 98], the magnitude of temperature differences was influenced by methodological choices, including measurement method, season, time of day and distance between points or areas compared. With similar variability, green roofs and vertical greening systems showed reductions in indoor air temperatures of up to 2.9 °C in Nigerian cities [67, 86] and roof temperature reductions of up to 9.2 °C in Durban (South Africa) [65]. In addition to temperature reductions from NbS actions, several studies provide qualitative evidence of urban residents, including those living in informal areas, using green spaces for temperature regulation [53, 99] and engaging in NbS actions such as backyard tree planting to adapt to urban heat [91]. Across these studies, reported effectiveness is modified by green space size, vegetation density, morphology, surrounding built form, and the presence of blue infrastructure.

Numerous studies report NbS contributing to flood risk reduction (*n* = 29) and increasing water availability (*n* = 17), primarily through River, catchment and drainage system NbS (figure 4), based mainly on qualitative evidence (table S6). Catchment management initiatives in Kenya [79, 80] and Ethiopia [100] contribute to addressing multiple climate hazards including flooding, drought, and landslides or soil erosion. These interventions reported reduced sediment transport of 25%–40%, increased streamflow and ground water levels, and reduced urban water treatment costs. Actions implemented include tree integration into agricultural systems, soil stabilisation, and the rehabilitation of riparian ecosystems. Other NbS interventions that contribute to reducing these risks include retrofitting stormwater ponds in Cape Town, which increased aquifer recharge potential by 83% [8, 101] and rainwater harvesting which reduced flood risk and increased water security in Nairobi (Kenya), Dar es Salaam and Bagamoyo (Tanzania) [54, 62]. Although figure 4 indicates relatively frequent reported success for flood risk reduction and water availability, much of this evidence is qualitative or based on project-level reporting rather than directly comparable hydrological metrics.

Evidence of NbS effectively addressing coastal risks was also largely qualitative (table S6). For example mangrove, sand dune and coastal wetland restoration and protection initiatives reported reduced exposure to sea level rise, and storm surges across multiple locations [60–63, 88, 102]. Where benefits were quantified, these were in terms of avoided costs. In the Tana Delta (Kenya), benefits of coastal flood risk reduction from mangrove ecosystems were valued at US$ 238–311 ha^−1^ yr^−1^ [64]. In Freetown (Sierra Leone), tree planting was projected to reduce the damages from landslides, floods, coastal erosion and sea level rise by almost US$ 40 million by 2050 [46].

Quantified mitigation outcomes were limited to carbon sequestration (table S6). Carbon stocks in urban trees and forests were estimated at 48–745 tC ha^−1^, varying by land use, tree density and location [73, 75, 103, 104]. Carbon sequestration rates were estimated at just over 1 tC ha^−1^ yr^−1^ for urban woodland in Bulawayo (Zimbabwe) [105], and up to 200 492 tCO_2_e for indigenous street trees in Tshwane (South Africa) over 30 years [96]. These sequestration rates are substantial but must be contextualised. They accumulate over decades of tree growth, maximum sequestration occurs during stand establishment (30–40 years), and carbon is released if trees are harvested or if vegetation is degraded. Urban forests thus provide modest but meaningful contributions to city-level mitigation while generating concurrent adaptation, biodiversity, and livelihood benefits.

While potential for greenhouse gas emission reductions exist in Green buildings [31], the achievement of mitigation through this mechanism is not reported in this corpus of NbS interventions. Studies in South African contexts indicate potential for meaningful energy reduction [65], but absolute values (kWh m^−2^ reduction) are less frequently reported than in global literature. However, no study that explicitly targeted GHG emissions reduction reported successful achievement of that outcome in the effectiveness coding. This represents a significant evidence gap for African cities with growing energy demand and electricity supply constraints.

Overall, the corpus provides substantive evidence that NbS can contribute to climate risk reduction and carbon sequestration in African cities. However, the diversity of intervention types, implementation scales and evaluation methods limits conclusions about the magnitude and comparability of these effects. Several studies also noted that it was too early to assess outcomes or that limited long-term monitoring constrained effectiveness assessment [30, 83, 106]. This highlights the need for longer-term monitoring, more consistent indicators and greater attention to health, hydrological and emissions outcomes in future evaluations.

### 3.5. Factors affecting feasibility

#### 3.5.1. Barriers and enablers

To provide evidence on the feasibility of NbS, including EbA, in African cities, we examined reported barriers and enablers of implementation across 112 studies, the stakeholders involved, and identified co-benefits, trade-offs and unintended outcomes. Across African cities, implemented NbS often combined place‐specific design, coalition building, and capacity investments that translated directly into delivery on the ground. These characteristics are consistent with the most frequently cited enabling conditions in our synthesis (figure 5). Specifically, multi‐stakeholder & cross‐sectoral collaboration (*n* = 46), public awareness & capacity (*n* = 41), and participatory planning & community engagement (*n* = 41) are components of more than a third of implemented NbS, with availability of financial support (*n* = 35) and data & information (*n* = 27) also being prominent enablers.

**Figure 5.**
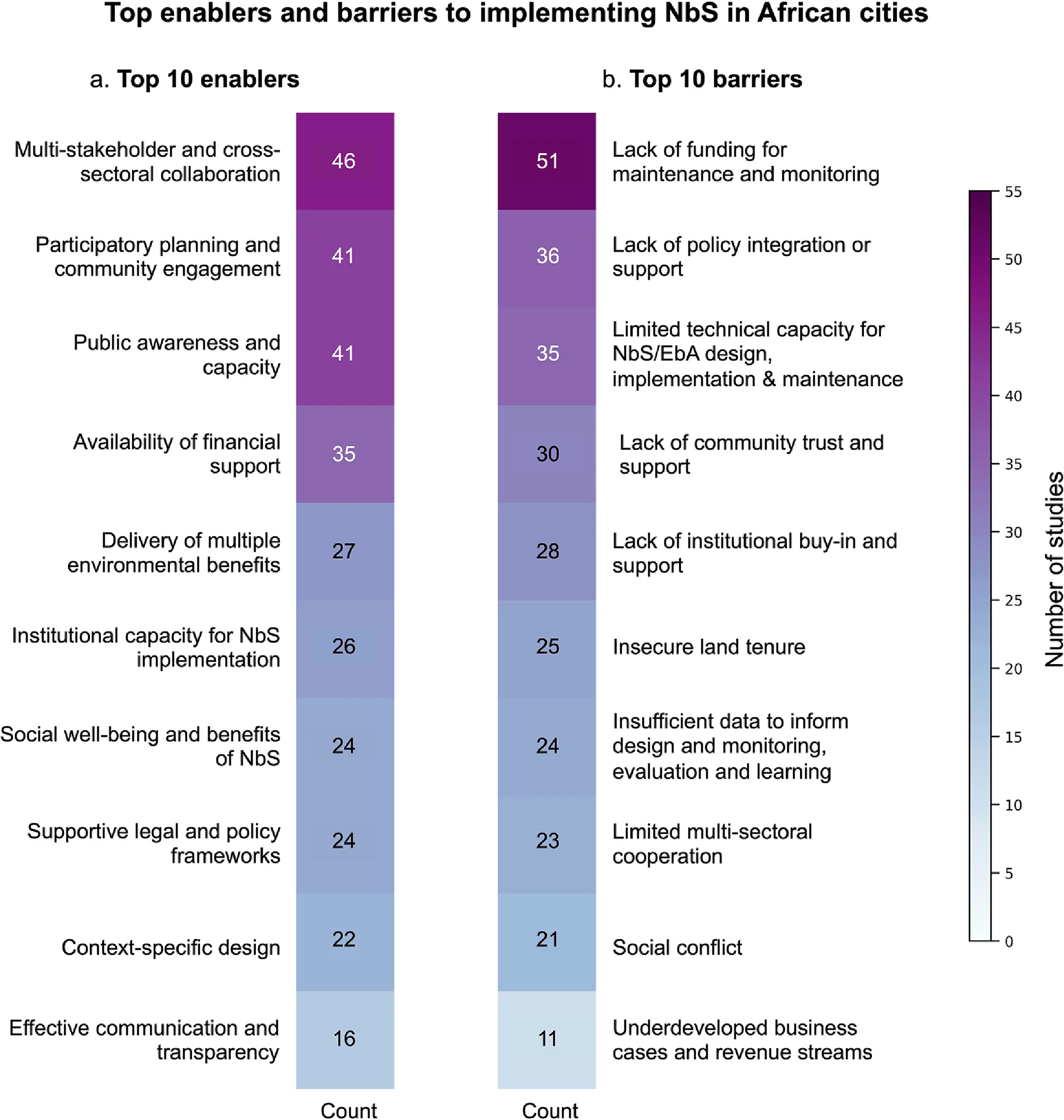
Most frequently reported enablers and barriers to implementing urban nature-based solutions (NbS) in African cities. Side-by-side heatmaps showing the ten most frequently reported enablers and barriers to implementing urban NbS in African cities. Panel a shows the top 10 enablers, aggregated across all NbS typologies. Panel b shows the top 10 barriers, also aggregated across all NbS typologies. Counts represent the number of studies (*n* = 112) in which each enabler or barrier was reported. The colour scale ranges from 0 to 55 studies in increments of five, enabling comparison between enablers and barriers. The most frequently reported enabler was multi-stakeholder and cross-sectoral collaboration, while the most frequently reported barrier was lack of funding for maintenance and monitoring.

Despite notable successes, implementers repeatedly flag inadequate funding/maintenance budgets (*n* = 51), limited technical capacity (*n* = 35), and insufficient data for monitoring, evaluation and learning (MEL) systems (*n* = 24) as constraints, alongside policy integration gaps (*n* = 36) and lack of community support (*n* = 30). Initiatives tended to fare better in cases where technical capacity was developed and responsibility for long-term maintenance was institutionalised [78, 82, 107]. For example, in Durban, an ecosystem restoration programme built capacity for identification and removal of alien invasive plants and integrated the maintenance of restored spaces into municipal functions and budgets [94]. The most frequently reported enablers and barriers both indicate that governance is a determining factor in the feasibility of NbS initiatives.

#### 3.5.2. Governance & socio‐political modifiers

Our analysis of stakeholder involvement across initiative phases (table S7) indicates that design and planning are most often led by local government, but typically co‐designed with residents/communities, local NGOs and CSOs, international agencies, and researchers, reflecting a distributed capability model that matches the place‐based nature of urban ecosystems. Implementation is even more community‐based. Residents, communities and local government dominate site works, with community leaders and local NGOs regularly providing the connective tissue for day‐to‐day delivery. International agencies and local governments are the most frequent funders, with national governments and private utilities less common. Direct community self‐funding appears, but at much lower frequency, which may reflect under-reporting of community funding in research outputs rather than a lack of investment. Together, these patterns underline that effective delivery hinges on aligning municipal mandates and finance with community stewardship and intermediary civil‐society capacity.

Consistent with reported enablers, multi-stakeholder participation emerges across the corpus, with varying levels of involvement across intervention phases, roles, and implementation contexts. Multi‐actor coalitions and participatory design are central to most projects, facilitated through collaboration (*n* = 46), public awareness and capacity (*n* = 41), and participatory planning and community engagement (*n* = 41). These governance ingredients recur in successful Green space, Rivers, catchments and drainage systems, Green building, and Urban agriculture and multi-use garden initiatives alike. Cape Town’s Liveable Urban Waterways programme illustrates the coalition model explicitly: multi-departmental city coordination across Catchment and Stormwater Management, Environmental Management, Resilience, Recreation and Parks, and Urban Planning is documented as central to successful integrated water-sensitive urban design, with the programme demonstrating simultaneous delivery of water security, flood management, and community livelihood outcomes [78]. Similarly, in Durban, river rehabilitation for climate adaptation and water security is governed through a partnership between eThekwini Municipality’s Environmental Planning and Climate Protection Department and the Water and Sanitation Unit, embedded within a broader multi-level governance arrangement spanning municipal, provincial, and national actors and involving non-state partners in design and monitoring [57]. In the Nalukolongo catchment in Kampala, community governance of SuDS is enabled through training of local leaders as advocates for blue-green infrastructure. In this case, effective community governance was found to depend on effective local training programmes, clearly defined stakeholder mandates, and collaborative platforms involving public, civil society, and community actors [59].

Community-based actors were the most prominent stakeholders across initiative phases (*n* = 172). Residents or community members were involved in 42% of planning processes (*P*), with participation increasing during implementation (*I*, 55%) and maintenance (*M*, 56%), indicating a central role in delivery and long-term management despite limited involvement as funders (9%). Community leaders demonstrated similarly consistent engagement across phases (*P* = 25%; *I* = 29%; *M* = 22%). However, they were not reported as funders in any cases, highlighting a clear separation between financial responsibility and governance roles. This pattern of participation is amplified in initiatives in informal settlements. Across the studies within informal areas (*n* = 20), residents or community members were involved in 70% of planning processes, 95% of implementation activities, and 85% of maintenance, emphasising the strength of local community governance systems in informal areas.

This level of participation enables NbS initiatives, including EbA, that require minimal financial expenditure but demand significant labour, organisation, and stewardship, including micro-scale greening, building-integrated prototypes and localised SuDS. Across informal settlements, these small‐footprint, low‐cost and community‐driven initiatives are most feasible where space is constrained and tenure is insecure. For example, an action research study on micro-SuDS in Diepsloot (Johannesburg) highlighted that they can be built for ‘almost no cost’ by using salvaged materials, illustrating how costs can be moved from financial capital to social organisation and local creativity [55]. However, such strategies require considerable time investment and are effective only at small scales.

Local government and municipal actors are the most consistently reported institutional stakeholders across NbS planning, implementation and maintenance in African cities, fulfilling a cross-departmental coordinating role. This is exemplified by Cape Town’s five-department Liveable Urban Waterways programme [78], Durban’s two-decade multi-level ecological infrastructure partnerships [47, 57, 82], and Lilongwe’s city-wide Ecological Corridor Initiative, whose implementation barriers expose the limits of top-down coordination without community alignment [56]. Municipal influence on building-integrated NbS operates primarily through regulatory frameworks rather than direct delivery.

National and regional governments showed relatively consistent involvement across intervention phases, reflecting their roles as funders (18% of studies) and oversight actors, despite slightly stronger engagement during planning stages. International organisations and development agencies were the most frequently reported funders of initiatives (38%), demonstrating their influence over the NbS implementation agenda through a strong association between international financing and initiative planning and design (*P* = 28%) rather than sustained involvement in long-term stewardship (*M* = 13%). These organisations include United Nations Agencies, development agencies and banks, transnational local government networks, international NGOs, and philanthropies. Similar frontloading trends, a reflection of co-design in project development and early implementation, were evident with local NGOs or CSOs (*P* = 29%; *I*= 16%; *M* = 13%) and researchers (*P* = 25%; *I* = 26%; *M* = 14%). The latter indicates a potential gap in long-term monitoring.

#### 3.5.3. Intervention costs

Funding limitations were the most frequently reported barrier to the longevity of NbS, including EbA, initiatives in this corpus, indicating that financing mechanisms play a significant role in determining feasibility. In some cases, international loans and grants flowed through national governments and development finance institutions, introducing complex procurement mechanisms that raised transaction costs and delayed implementation [62, 88]. Donor dependency and the inability to scale financing beyond pilot projects remain among the most consistently cited barriers to NbS mainstreaming across the corpus [26, 61, 108].

Despite financial barriers being frequently identified, most studies do not provide comprehensive financial data. Indicative cost data points in the corpus span several orders of magnitude, reflecting the diversity of intervention types and institutional scales involved. Reports of the actual costs of implemented programmes vary widely depending on the scale, nature of the work and context, even within a single city. Across Cape Town’s Liveable Urban Waterways Programme, capital expenditure ranged from approximately US$26 400 (R450 000) for the Skilpadsvlei wetland rehabilitation to US$3.5 million (R60 million) for the construction of Green Point Urban Park, with the overall programme representing more than R100 million of public investment [78]. This spread reflects differences in intervention type, site complexity, and engineered infrastructure [78]. This cost variability is not unique to Cape Town. A review of 181 urban tree planting projects across African cities estimated a per-tree planting cost of between US$10 and US$100, but found that a shortage of public data, inconsistent monitoring frameworks, and limited post-planting evaluation make systematic cross-case comparison extremely difficult [76]. These data points illustrate the range of investment that NbS, including EbA, can involve, but they are not sufficient to establish reliable, transferable benchmarks for the continent. Cost boundaries, including costed items, timescales, and the apportionment between NbS and non-NbS components, are not defined consistently across studies, and Nilsson *et al* [54] explicitly note that the transferability of cost results to other solutions and contexts remains limited. This is a constraint that echoes the broader data shortage and inconsistent monitoring documented across urban tree planting and other NbS projects in African cities [76].

Even when grant funding is available, a lack of sufficient technical studies at the outset can lead to unrealistic budgets. In Tanzania, for example, the implementation of a large-scale coastal restoration project was hindered by insufficient funding, overly ambitious budgetary targets, and the lack of detailed engineering studies and costing at the design stage. Delays between budgeting and implementation exacerbated the problem [62, 83].

Funding for monitoring, maintenance, and long-term stewardship present a common challenge to the sustainability of implemented initiatives. The Upper Tana-Nairobi Water Fund estimated a requirement of US$1 million per year over ten years to implement catchment rehabilitation across 50 000 farms, with an endowment fund (target US$7.5 million) established to sustain operations and maintenance once initial investment pressure eases [79]. Even this relatively well-capitalised case highlights the challenge of ongoing threats from invasive species and ecosystem degradation increasing maintenance costs. To support business cases for NbS implementation, including EbA, some studies quantified ecosystem service values. For example, in urban woodland in Bulawayo (Zimbabwe) values were estimated at US$340–490 ha^−1^ yr^−1^ for fuelwood and US$4.04 ha^−1^ yr^−1^ for carbon sequestration [105]. These values establish that NbS can generate economic returns, though returns are modest relative to capital investments, requiring sustained financing over decades to recover capital. Municipal, private sector and utility funding of maintenance can significantly support long-term sustainability.

#### 3.5.4. Trade-offs and unintended outcomes

Trade-offs relating to land availability and use were recorded in 37% of studies (*n*= 41), which frequently mentioned development priorities or perceived better land use being at odds with restoration or continued protection of green spaces. This reflects the significant opportunity cost of NbS initiatives, including EbA. For example, for the restoration of mangroves in the Tana Delta (Kenya), the opportunity cost (US$774 ha^−1^ yr^−1^) was estimated as an order of magnitude greater than the maintenance cost (US$59 ha^−1^ yr^−1^) [64].

Even within managed and restored ecosystems, access trade-offs persist. Immediate provisioning uses of green space such as harvesting fuelwood, wild plants and fruits can conflict with the long-term contributions those spaces are intended to deliver, including heat mitigation and stormwater attenuation [99, 105]. More broadly, ecological rehabilitation programmes, like those in the Sahel, can actively increase the vulnerability of the poorest communities through land tenure losses, restricted livestock mobility, and elite capture of rehabilitated land [109]. At the watershed scale, both the Minzir micro-watershed project in Ethiopia and the Naivasha basin in Kenya illustrate the distributional trade-offs that accompany catchment-level NbS interventions [80, 100]. Resistance and disruption to livelihoods and everyday uses of open space appear to be systemic rather than exceptional across NbS programmes in urban Africa, encompassing the displacement of livelihood activities and community uses from sites undergoing formalisation [110]. Other reported trade-offs are dominated by financial and maintenance costs (*n* = 35), time lags between implementation and realised benefits (*n* = 26), and uncertainty in quantifying multi‐benefit outcomes (*n* = 25).

Almost one third (*n* = 35) of included studies reported on disservices or unintended outcomes of NbS initiatives, including EbA. The most frequently recorded categories include increases in pests and invasive species which increased maintenance costs (*n* = 10), crime and safety risks (*n* = 10), pollution from sewage or solid waste (*n* = 7), human health risks from pathogens (*n* = 7), human–wildlife conflict (*n* = 7), dust or soil erosion (*n* = 5), damage to infrastructure by plants or animals (*n* = 4), and increased water consumption and drought risk (*n* = 6). Reduced aesthetic value (*n* = 4) and occasional increases in flood (*n* = 3) or wildfire risk (*n* = 2) are also noted. These disservices cluster by context for example, riparian and wetland corridors more often note pathogen risks [55] and reduced aesthetics where stagnation or sewage ingress occur, while urban forests and public‐realm plantings more often note safety concerns, invasive species or pest issues, root damage, and wildlife encounters [90].

## 4. Discussion

This continental synthesis supports three key conclusions. First, NbS, including EbA, are already widely used in African cities to respond to climate risk, although documented implementation is concentrated in a few large cities and smaller urban centres remain under-represented. Second, NbS in African cities are valued as much for what they do for livelihoods as for their role in reducing the harmful impacts of climate change. Adaptation outcomes are reported far more often than mitigation outcomes, and livelihood security is frequently reported alongside heat and flood risk reduction. This reflects contexts in which income, food and water insecurity are themselves drivers of vulnerability to climate change. Third, while individual NbS interventions in African cities are generally familiar to NbS practice elsewhere, what is distinctive about the African urban context is how they are combined and the conditions under which they are delivered. Residents and community actors provide much of the labour of implementation and stewardship, especially in informal settlements where state infrastructure and formal land-use management are limited. International agencies are the most frequently reported funders, but their involvement is concentrated in planning and design rather than long-term stewardship. Moreover, rapid urban growth intensifies competition for well-located land, creating trade-offs between NbS and other urban land uses. Under these conditions, governance, long-term finance and the distribution of benefits and burdens, rather than technical design alone, determine whether NbS reduce vulnerability or redistribute it.

We synthesised evidence on implemented NbS, including EbA, from 112 studies across 134 African urban areas and 42 countries, demonstrating widespread adoption of nature-based strategies for urban climate action on the continent. This evidence shows that NbS strategies are applicable across scales, from individual settlements to multi-city networks or catchments, and that they contribute to reducing climate risks across these scales. NbS, and particularly EbA, are being used in African cities to address climate risk in vulnerable low-income areas, including informal settlements, with a focus on heat mitigation, flood risk reduction, and improved livelihood security. However, this evidence is predictably clustered in a handful of mega-cities and focuses on well-known informal settlements like those in Kibera (Nairobi) and Khayelitsha (Cape Town). While widespread, evidence in secondary cities and towns is frequently limited to one or two studies. Funding geographies for implementation of NbS in Africa centre on well-established international organisations and large metropolitan governments. Similar geographies of research funding concentrate the evidence base on well-established institutions and researchers. Our inclusion of only English language documents and reliance on digital archiving for access to grey literature may have led to the exclusion of evidence from secondary cities, Central Africa and parts of West Africa, where other adaptation reviews have also found evidence gaps [2, 111].

The five-class typology developed in this review illustrates how NbS strategies for responding to climate change in African cities build on, adapt and extend existing practices. These NbS strategies work with ecosystems and green-blue infrastructure including recreational green spaces, urban and peri-urban food production, hybrid grey-green stormwater management, building-integrated greening, and coastal ecosystem restoration. The typology departs from many Global North-oriented frameworks [39] by foregrounding the empirical configurations observed in African urban contexts: Green spaces; Rivers, catchments and drainage systems; Coastal systems; Green buildings; and Urban agriculture and multi-use gardens. It is therefore intended not as a universal classification, but as an Africa-grounded synthesis tool that reflects implemented NbS reported in both peer-reviewed and grey literature, including actions in informal settlements and secondary cities that are often marginal in global NbS evidence bases [8, 10].

The distribution of studies across the typologies and outcomes captures the research landscape on NbS for climate action, but not necessarily the full practice of NbS across African cities. For example, literature on urban agriculture in Africa does not consistently address climate benefits [112] and small-scale privately implemented NbS integrated into buildings or gardens may not be fully reported in digitally archived literature. The concentration of evidence on heat mitigation may also reflect that this is easier to measure and attribute to NbS than mitigation of other hazards like coastal erosion or storm surges. Evidence for the other four typology classes is thinner, and their hydrological, coastal and livelihood outcomes are mostly reported qualitatively (section 3.4). Shared indicators for these outcomes, reported alongside the scale of intervention and the duration of monitoring, would allow these typologies to be evaluated with greater confidence and would help to reveal outcomes that currently go undocumented.

The majority of the reviewed evidence confirms that urban NbS, including EbA, across the continent effectively provide multiple benefits, including carbon sequestration, adaptation, livelihoods and social uses, and enhanced ecosystem health and biodiversity. In this way it shows the potential of NbS to address underlying drivers of vulnerability to climate change, such as livelihood precarity, and to reduce exposure to climate hazards. Our review also illustrates the potential synergies between biodiversity and climate action through NbS, with almost half of the urban areas where NbS have been implemented being within global biodiversity hotspots. In some cases, this pattern reflects the strategic prioritisation of interventions that address multiple planetary crises simultaneously. This review provides a practice-relevant synthesis of empirical evidence of NbS for climate action in African cities, thereby contributing to bridging the theory-practice gap prevalent within the field [113].

As urban populations grow across the continent, there is increasing pressure to develop well-located land in African cities for housing and economic uses. Trade-offs between development and the protection and restoration of land for NbS, including EbA, were reported in 37% of the studies we reviewed. Managing these land-use trade-offs equitably depends on collaboration among multiple stakeholders, from local governments and traditional authorities responsible for land use management, to residents, local CSOs/NGOs and sometimes private sector actors involved in the day-to-day management of ecosystems and ecological infrastructure [44, 53, 78]. The involvement of these actors is frequently contingent on the demonstrated effectiveness of ecosystems and green-blue infrastructure in delivering adaptation and people-centred benefits and on strong coalitions sustaining cooperation across sectors and agencies and building awareness, support and capacity for NbS implementation. In informal settlements, where the absence of state-provided infrastructure and formal land-use management often exacerbate vulnerability to climate risks, the dependence of effective NbS initiatives, including EbA, on community engagement is particularly marked.

International development organisations and financiers, along with some national governments have supported the uptake of NbS in African cities through funding and involvement in project design. However, international agendas associated with this finance are not always aligned with local needs and priorities [26, 114]. In addition, project cycles are short relative to timelines for ecosystem establishment, and dependence on external support frequently leaves gaps in long-term maintenance and monitoring which local and city-level actors struggle to fill. This review confirms the persistence of the financing gap, with limited evidence in this literature on the types of transferable, context-adapted financing models that could exist to effectively support NbS in African cities. Isolated examples indicate that long-term viability is achievable, but these are outliers rather than reproducible templates. Systematic analysis of financing mechanisms suited to informality and sub-municipal governance structures remains a priority for enabling scaled NbS investment across the continent.

While this systematic review shows widespread implementation of NbS, it relies on self-reported outcomes and effectiveness data and reflects the limited quantification of adaptation and mitigation benefits. This is partly a result of limited longitudinal monitoring and a bias towards studies that report soon after implementation. Few studies in this corpus reported clear and comprehensive cost estimates for the implementation and maintenance of NbS initiatives and none provided estimates of the efficacy and feasibility of implementing NbS, including EbA, under future climate scenarios. Similarly, studies on heat mitigation report reductions in air and surface temperatures rather than in heat stress. Attributing changes in heat-related illness or mortality to NbS would require dedicated epidemiological research. Such research is needed to establish the health value of urban cooling through NbS in African cities.

The Introduction identifies distributional trade-offs and the risk that NbS, including EbA, may inadvertently reinforce existing vulnerabilities as a key gap in regionally-grounded evidence. This is not unique to NbS, such risks are well documented across climate change adaptation and development assistance. Interventions framed as technical or incremental have often reproduced unequal structures and, in some cases, deepened marginalisation by failing to address underlying drivers of vulnerability [115]. Our findings show that NbS, even when used for climate change adaptation (EbA), are not exempt from the trade-offs that are increasingly documented in the adaptation literature, and that have a long history in critiques of development assistance. We offer a partial but sobering response: while low-income communities are the most frequently identified target beneficiaries (*n* = 35) and youth and women participate substantially through green jobs and stewardship roles, 23 studies document livelihood disruption, displacement of informal traders, or reduced access to land and grazing. These distributional impacts are concentrated in contexts where NbS are implemented without secure land tenure, adequate community consultation, or benefit-sharing arrangements. The evidence does not yet support confident conclusions about whether urban NbS in African cities reliably reduce rather than redistribute vulnerability. To fill this gap, future research needs to explore the underlying causes of inequitable outcomes of NbS as well as focus on longitudinal and household-level monitoring as a condition of responsible scaling of NbS, including EbA.

Several limitations should be considered when interpreting these findings. First, the review captures documented implementation rather than all NbS practices, and therefore reflects uneven publication, reporting and digital archiving across countries, cities and organisations. Second, because the corpus spans multiple disciplines, methods and intervention scales, we synthesised reported outcomes narratively rather than through meta-analysis. Third, many studies report outcomes soon after implementation and rely on project- or author-reported evidence, likely leading to positive reporting bias and limiting inference about long-term effectiveness. Fourth, although we included transparency and validity indicators in data extraction, we did not exclude studies through quality appraisal to avoid systematically removing grey literature and locally grounded evidence. These limitations mean that the review is strongest as a synthesis of documented implementation, outcome pathways and feasibility conditions, rather than as a continent-wide quantitative estimate of NbS effectiveness.

## 5. Conclusions

This review provides the first continental synthesis of studies on implemented NbS, including EbA, in African cities. In doing so, it addresses a gap in synthesised evidence on which NbS are implemented, their reported effectiveness, and the factors that shape their feasibility, including in informal settlements. Implementation is widespread across the continent, although the evidence is concentrated in a few large cities. Reported outcomes are predominantly for adaptation, delivered by reducing hazard impacts, chiefly heat and flooding, and by strengthening livelihood security, while mitigation outcomes are largely limited to carbon sequestration by urban green spaces. Evidence of effectiveness is positive but mostly qualitative and short-term. Green spaces dominate the evidence, and many initiatives combine intervention classes to deliver adaptation, social and biodiversity benefits together. For policy and practice, the central implication is that NbS, including EbA, should be mainstreamed into urban planning, service delivery and infrastructure investment rather than treated as stand-alone environmental or climate projects.

Feasibility depends less on intervention type than on governance and finance. Multi-stakeholder collaboration anchored by local actors and meaningful community participation are the most consistent enablers, while insufficient long-term funding for maintenance and monitoring is the most frequently reported barrier. At the same time, documented instances of livelihood displacement, land-use trade-offs, and governance exclusion in informal settlements mean that positive climate outcomes cannot be assumed to reach the most exposed urban residents without deliberate equity safeguards, as is the case with other adaptation options. Equity considerations should therefore be treated as part of NbS design rather than as downstream social co-benefits. To that end, national adaptation plans and urban climate finance instruments that promote NbS should also resource long-term maintenance and monitoring and build in these safeguards. Priority research gaps include longitudinal and fine-scale effectiveness monitoring under current and projected climate conditions, financing mechanisms suited to informal settlements and linking formal and informal contexts, systematic assessment of the reasons for inequitable outcomes and of who bears the costs and who captures the benefits of NbS investment, and evidence from smaller cities and under-represented regions. Addressing these gaps is a prerequisite for translating the continental adoption documented here into equitable and durable urban climate resilience.

## Data Availability

The data that support the findings of this study are openly available at the following URL/DOI: https://doi.org/10.25375/uct.31563052 [116]. Supplementary figures & tables available at: https://doi.org/10.1088/1748-9326/aeb0ba/data1.
